# Draft Genome Sequence of the Polyextremophilic *Exiguobacterium* sp. Strain S17, Isolated from Hyperarsenic Lakes in the Argentinian Puna

**DOI:** 10.1128/genomeA.00480-13

**Published:** 2013-07-25

**Authors:** Omar F. Ordoñez, Esteban Lanzarotti, Daniel Kurth, Marta F. Gorriti, Santiago Revale, Néstor Cortez, Martin P. Vazquez, María E. Farías, Adrian G. Turjanski

**Affiliations:** Laboratorio de Investigaciones Microbiológicas de Lagunas Andinas (LIMLA), Planta Piloto de Procesos Industriales Microbiológicos (PROIMI), CCT-CONICET, Tucumán, Argentinaa; Instituto de Biología, Molecular y Celular de Rosario (IBR-UNR-CONICET), Rosario, Argentinab; Facultad de Ciencias Exactas y Naturales, Universidad de Buenos Aires, Buenos Aires, Argentina, and Instituto de Agrobiotecnología Rosario (INDEAR)-CONICET, CCT-CONICET, Rosario, Argentinac; Departamento de Química Inorgánica, Analítica y Química Física/INQUIMAE-CONICET, Facultad de Ciencias Exactas y Naturales, Universidad de Buenos Aires, Ciudad Universitaria, Buenos Aires, Argentinad; Departamento de Química Biológica, INQUIMAE-CONICET, Facultad de Ciencias Exactas y Naturales, Universidad de Buenos Aires, Ciudad Universitaria, Buenos Aires, Argentinae

## Abstract

*Exiguobacterium* sp. strain S17 is a moderately halotolerant, arsenic-resistant bacterium that was isolated from Laguna Socompa stromatolites in the Argentinian Puna. The draft genome sequence suggests potent enzyme candidates that are essential for survival under multiple environmental extreme conditions, such as high levels of UV radiation, elevated salinity, and the presence of critical arsenic concentrations.

## GENOME ANNOUNCEMENT

The high-altitude Andean lakes (HAAL) consist of several shallow lakes located in a high-altitude desert known as Puna. They are exposed to extreme environmental conditions such as high levels of UV radiation, elevated salinity, and the presence of heavy metals and metalloids, mainly arsenic (1–5). *Exiguobacterium* was identified as one of the dominant Gram-positive taxa in HAAL (5). *Exiguobacterium* spp. have been found in a wide range of habitats, including cold and hot environments with temperatures ranging from -12 to 55°C (6, 7). This fact confers substantial interest in the genus as a potential model system for the investigation of attributes that may correlate with adaptation and evolution of organisms to diverse thermal regimens (8). Here, we present the draft genome sequence of *Exiguobacterium* sp. strain S17, which was isolated from a stromatolite placed in Laguna Socompa, northern Argentina, at the HAAL (9).

The genome sequence was obtained using a whole-genome shotgun (WGS) strategy with a 454 GS Titanium pyrosequencer at the Instituto de Agrobiotecnología Rosario (INDEAR), Argentina. Assembly was done using 454 Newbler version 2.5.3 using the -urt option with 63× genome coverage. This assembly generated 193 large contigs. The draft genome was 3,139,227 bases in length, with a mean G+C content of 53.14%. Genome annotation was done using the standard operating procedures (SOPs) for prokaryotic annotation from ISGA (10) and from the RAST annotation server (11). A total of 3,218 coding sequences (CDSs) and 49 structural RNAs (48 tRNAs) were predicted. Annotation covered 360 RAST subsystems (43%) with 1,381 CDSs, while 1,149 CDSs (36%) were classified as hypothetical proteins. The complete 16S rRNA gene presented a maximum identity of 98.9% with that of *Exiguobacterium aurantiacum* strain DSMZ 6208.

The genome of *Exiguobacterium* sp. S17 presented 102 genes devoted to the stress response according to RAST (11), a greater number than the one observed in the previously sequenced *Exiguobacterium* genomes (86 genes in *Exiguobacterium sibiricum* [12], 70 genes in *Exiguobacterium antarticum* B7 [13], and 67 genes in *Exiguobacterium* sp. AT1b [14]). Strain S17 contains a complete DNA repair system, including UvrABC, MutL-MutS, and bacterial photolyase, and several genes related to resistance to toxic compounds, such as antibiotics, arsenic, cadmium, and mercury. The high resistance to arsenic previously observed in S17 can be explained based on the greater number of genes reported to detoxify this compound, which is 7 genes in comparison to 4 genes observed in *E. sibiricum* and 5 genes each in *E. antarticum* B7 and *Exiguobacterium* AT1b. A striking difference between S17 and other *Exiguobacterium* spp. is the presence of the *acr3* gene, which is known to be a contributor to cell detoxification against arsenite, one of the most toxic arsenic species. This is the first report of the presence of the *acr3* gene in this genus.

This genome reveals essential adaptations for survival under multiple extreme environmental conditions and is an attractive model to study novel mechanisms of tolerance to extreme environmental factors, allowing for the identification of new systems exploitable for the bioremediation of metals and metalloids.

### Nucleotide sequence accession numbers.

This whole-genome shotgun project has been deposited at DDBJ/EMBL/GenBank under the accession no. ASXD00000000. The version described in this paper is the first version, accession no. ASXD01000000.
